# Diverse Molecular Genotypes of* Mycobacterium tuberculosis* Complex Isolates Circulating in the Free State, South Africa

**DOI:** 10.1155/2016/6572165

**Published:** 2016-03-17

**Authors:** Anneke Van der Spoel van Dijk, Pakiso M. Makhoahle, Leen Rigouts, Kamaldeen Baba

**Affiliations:** ^1^Department of Medical Microbiology, Faculty of Health Sciences, University of the Free State, Bloemfontein, South Africa; ^2^National Health Laboratory Service, Universitas Academic Laboratory, Bloemfontein 9301, South Africa; ^3^Unit for Drug Discovery Research, Department of Health Sciences, Biomedical Technology, Faculty of Health and Environmental Sciences, Central University of Technology, Bloemfontein 9300, South Africa; ^4^Mycobacteriology Unit, Institute of Tropical Medicine, Nationalestraat 155, 2000 Antwerp, Belgium; ^5^University of Antwerp, 2000 Antwerp, Belgium

## Abstract

Tuberculosis is a serious public health concern especially in Africa and Asia. Studies describing strain diversity are lacking in the Free State region of South Africa. The aim of the study was to describe the diversity of* Mycobacterium tuberculosis* (*M. tuberculosis*) strain families in the Free State province of South Africa. A total of 86* M. tuberculosis* isolates were genotyped using spoligotyping. A 12-locus mycobacterial interspersed repetitive units-variable-number tandem repeats (MIRU-VNTRs) typing was used to further characterize the resulting spoligotyping clusters. SITVITWEB identified 49 different patterns with allocation to six lineages including Latin-American-Mediterranean (LAM) (18 isolates), T (14 isolates), Beijing (five isolates), S (six isolates), Haarlem (one isolate), and X (five isolates), while 37 (43.0%) orphans were identified. Eight clusters included 37 isolates with identical spoligotypes (2 to 13/cluster). MIRU-VNTR typing further differentiated three spoligotyping clusters: SIT1/Beijing/MIT17, SIT33/LAM3/MIT213, and confirmed one SIT34/S/MIT311. In addition, SpolDB3/RIM assignment of the orphan strains resulted in a further 10 LAM and 13 T families. In total, LAM (28 isolates) and T (27 isolates) cause 63% of the individual cases of MTB in our study. The Free State has a highly diverse TB population with LAM being predominant. Further studies with inclusion of multidrug-resistant strains with larger sample size are warranted.

## 1. Introduction 

Tuberculosis (TB) still remains a public health challenge especially in the African region where 28% of the estimated 9.6 million cases were reported in 2014 [1]. South Africa is one of the countries with the highest incidence of TB (834 cases per population of 100 000 in 2014) [1]. The case load for the Free State province, South Africa, was reported as 17710 in 2014 and the province has one of the least cases of MDR (3%) compared to other provinces (20%) [2].

The introduction of molecular epidemiology has greatly improved the understanding of the TB transmission patterns and genetic diversity of* Mycobacterium tuberculosis* (MTB) strains in different geographical locations [3, 4]. There are currently three main genotyping methods including IS6110-restriction fragment length polymorphism (IS6110-RFLP), spacer oligonucleotide typing (spoligotyping), and mycobacterial interspersed repetitive units-variable-number tandem repeats (MIRU-VNTRs) [4, 5]. MIRU-VNTRs are based on PCR amplification of genetic elements named MIRU that are located mainly in intergenic regions dispersed throughout the MTB genome. Each MIRU generates fragments of different sizes for different strains and the number of repeats at each locus can be determined [6]. MIRU-VNTR typing is fast and highly reproducible genotyping method and it can be performed by amplifying a panel of 12, 15, or 24 loci [6, 7]. The discriminatory power of the MIRU-VNTR assay is proportional to the number of loci evaluated. The combination of MIRU-VNTR typing with spoligotyping has shown a discriminatory power close to the IS*6110-*RFLP typing [8].

In South Africa most of the genotyping studies were done in provinces with high multidrug-resistant strains (MDRs) such as Western Cape [9, 10], Gauteng [11, 12], and KwaZulu-Natal [13, 14]. However, little data is available from most of the provinces of South Africa, especially the Free State region with low burden of MDR-TB [15, 16]. The purpose of this study was to determine the MTB strain types circulating in Free State using spoligotyping and MIRU-VNTR typing (original 12 loci).

## 2. Materials and Methods

### 2.1. Study Site and Sample

The Free State population consists of mainly three densely populated districts: Lejweleputswa, Mangaung, and Thabo Mofutsanyane. A convenience sample of 86 DNA extracts of MTB isolates available was included in the study. All strains were originally isolated on Löwenstein Jensen (LJ) slants and drug susceptibility testing was determined using the proportion method on LJ slopes.

Genomic DNA was extracted using a phenol-chloroform method as previously described [9]. DNA concentrations were determined by spectrophotometry using a NanoDrop ND-100 Spectrophotometer v3.01 (NanoDrop Technologies Inc., Wilmington, US). Ethical approval (114/06) to conduct the study was obtained from the Ethics Committee of the Faculty of Health Sciences, University of the Free State, Bloemfontein, South Africa.

### 2.2. Spoligotyping

Spoligotyping was performed using the commercially available kit (Isogen Bioscience BV, Maarssen, The Netherlands) according to the manufacturer's instructions. The results were recorded in a binary and octal format representing the 43 spacers.

### 2.3. MIRU-VNTR Typing

Selected MTB isolates belonging to (SIT) 1, 33, and 34 were further analyzed using the 12-locus MIRU-VNTR typing. The 12-locus MIRU-VNTRs consist of loci 154 (MIRU02), 580 (MIRU04 or ETRD), 960 (MIRU10), 1644 (MIRU16), 2059 (MIRU20), 2531 (MIRU23), 2687 (MIRU24), 2996 (MIRU26), 3007 (MIRU27 or QUB5), 3192 (MIRU31 or ETRE), 4348 (MIRU39), and 802 (MIRU40) [6]. Loci were individually amplified using primers and methodology as described elsewhere [6]. The amplicons were separated in 3% agarose gel (Whitehead Scientific Pty. Ltd., Cape Town, SA) using 100 bp ladder (New England Bio-Labs Inc., Hitchin, UK) as the size marker. Results from each of the 12 loci were combined into a numerical allelic profile.

### 2.4. Strain Classification and Phylogenetic Analysis

All genotyping data were entered into a Microsoft Excel sheet. The spoligotyping patterns in octal format and 12-locus MIRU-VNTR profiles were compared to an updated SpolDB4 database (http://www.pasteur-guadeloupe.fr:8081/SITVITDemo/) [17] SITVIT2 of Pasteur Institute of Guadeloupe available on the SITVITWEB (http://www.pasteur-guadeloupe.fr:8081/SITVIT_ONLINE/) [18], which compared spoligotyping data at the time of analyses to genotyping information of more than 75 000 MTB strains [19]. SITVITWEB provides SIT and MIRU International Type (MIT) numbers or orphan status to uploaded strains. When two or more patient isolates were present in the database with identical profiles, a SIT or MIT number was assigned and if not, it was deemed an orphan strain. Lineages and sublineages were assigned to strains according to the supplemental updated SpolDB4 profiles (http://www.pasteur-guadeloupe.fr:8081/SITVIT_ONLINE/) [18]. Strains were further assigned TB-lineage and probable families and subfamilies using TB-insight: TB-lineage and SPOTCLUST according to the SpolDB3 model by applying rules for the presence or absence of specific spacers in a specific order combined with the Randomly Initialised Model (RIM) [20]. TB-lineage assigns a lineage based on the seven Centres for Disease Control and Prevention (CDC) approved major genetic groups divided into modern (East-Asian or Beijing, Euro-American, and East-African Indian) and ancestral (Western African 1 and Western African 2 representing* M. africanum*,* M. bovis, *and Indo-Oceanic) MTB types [21]. The genetic relationship of the isolates was demonstrated using the MIRU-VNTRplus database and spoligotyping data to draw a phylogenetic tree employing Jaccard's coefficient to calculate the distance matrix and the neighbour-joining clustering algorithms (NJ) rooting from a* M. canettii *(*M. prototuberculosis*) strain to create the dendogram [22].

### 2.5. Multiplex Polymerase Chain Reaction (PCR) Analysis

Five isolates identified as Beijing strains by SITVIT2 were evaluated by multiplex PCR to identify the presence of W type Beijing strains by detecting a direct repeat of IS*6110* with a 556 base pair (bp) intervening sequence (NTF-1) [23]. Amplicons were analyzed by electrophoresis on a 2% Low Melting agarose gel (BioWhittaker molecular applications, USA) at 100 V for 2 h. The gel was stained with 0.5 *μ*g/mL ethidium bromide and photographed using Uvipro (Whitehead Scientific Pty. Ltd.) system.

## 3. Results

DNA extracts from 86 clinical MTB isolates and an H37Rv control strain were analyzed using spoligotyping. Three of eight resulting spoligotyping clusters were further analyzed using 12-locus MIRU-VNTR typing. The study population included 52 (60%) males and 33 (38%) females, while the gender of one patient was not available. Twenty-three (27%) of the isolates were from patients in the Lejweleputswa district, 22 (26%) from Thabo Mofutsanyane, and 41 (47%) from Mangaung.

The genotyping results are summarized in Table 1. Spoligotyping grouped 49/86 (57%) of the strains into SIT-shared international types representing six lineages. The Latin-American-Mediterranean (LAM) was the most prevalent containing 18/86 isolates (20.9%), followed by T 14/86 isolates (16.3%), S 6/86 isolates (7.0%), X 5/86 isolates (5.8%), Beijing 5/86 isolates (5.8%), and Haarlem (H) 1/86 isolates (1.2%) giving a clustering rate of 33%. However, 37/86 (43.0%) isolates were not described before and regarded as orphans.

**Figure 1:**
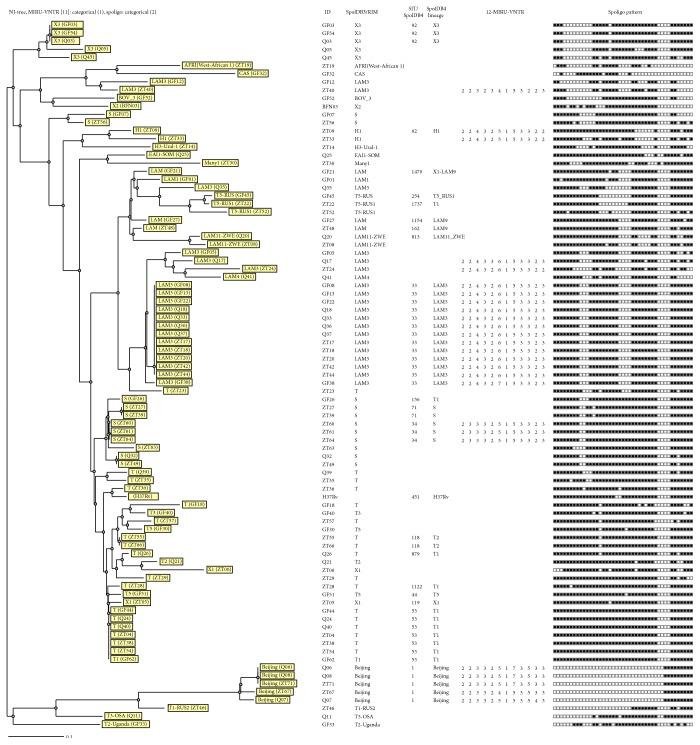
MIRU-VNTRplus cluster analysis of 86 isolates and an H37Rv control strain with spoligotyping and MIRU-VNTR profiles investigated in this study. The phylogenetic tree was rooted from* M. canettii* arranged according to similarities of spoligotypes using a Jaccard distance coefficient of 2 and for MIRU-VNTR types using a categorical value of 1. Included in the figure is, from left, phylogenetic tree drawn using neighbour-joining clustering algorithms (NJ), strain ID, most probable lineage determined with the TB-lineage database (SPOTCLUST according to the SpolDB3, combined with the Randomly Initialised Model (RIM)), SIT number and lineage according to SpolDB4 (MIRU-VNTRplus DB version), MIRU-VNTR profiles, and spoligotypes (1 to 43).

Three of the clusters, SIT1/Beijing (*n* = 5), SIT33/LAM3 (*n* = 13), and SIT34/S (*n* = 3), were further analyzed using 12-locus MIRU-VNTR typing. One of the clusters was differentiated into two MIRU international types (MIT): clone SIT1/Beijing/MIT17 (three isolates), and two isolates differed by two loci (Figure 1). Likewise, SIT33/LAM3/MIT213 clustered 12 isolates and SIT34/S/MIT311 (2 isolates).

**Figure 2:**
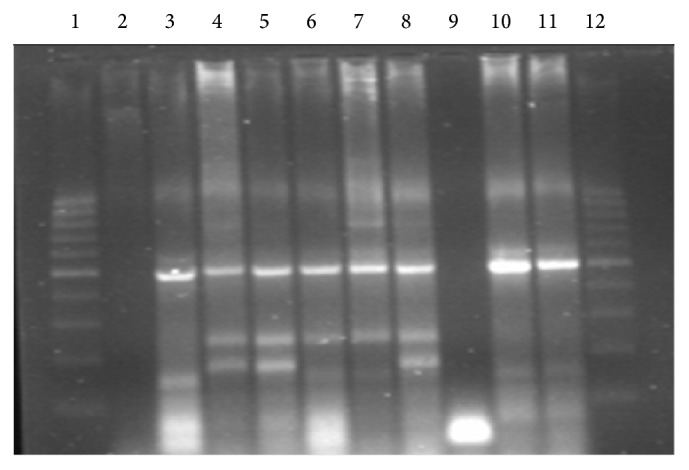
Amplification products from multiplex PCR analysis for Beijing strains of* M. tuberculosis*. Lanes 1 (100 bp DNA ladder), 2, and 9 (negative control and amplification control), lanes 4 (Q08), 5 (Q07), 6 (Q06), 7 (Q06), and 8 (ZT71) contain three-fragment pattern characteristic for strain W and lanes 3 (ZT67), 10 (ZT67), and 11 (H37Rv) contained only two fragments generated by internal positive control primers.

Other clusters identified were SIT92/X1 (three isolates), SIT53/T1 (seven isolates), SIT118/T2, and SIT71/S (two isolates). The last cluster contained two isolates identified as orphans using SITVITWEB, but probably belonging to lineage S (TB-insight: TB-lineage analysis). The three SIT1/Beijing/MIT17 isolates were confirmed as W types by multiplex PCR (Figure 2) that confirmed the presence of the ntf-1 intervening sequence [23].

All orphan strains were assigned the most probable lineage and sublineage using the TB-insight database option TB-lineage and SPOTCLUST with the SpolDB3 combined with the RIM model classification (Table 1). This analysis classified 29 more isolates into the two main families LAM (LAM3 (six isolates), other LAM types (four isolates)) and T (14 isolates). Thus the number of isolates belonging to LAM and T increased to 28 and 27, respectively, resulting in 64% (55/86) of the individual cases of MTB in our study. The spoligotype diversity within each of these families varied substantially, with LAM consisting of only one LAM3 clone (SIT33/LAM3/MIT213 with 12 isolates) and the remaining 16 in other LAM subfamilies.

Within the T family, 23/27 (85%) were assigned to the T1 subfamily, and the remaining 5/27 (18%) belonged to other T subfamilies.

Other families with more isolates identified with TB-lineage, such as the S family (a total of 11 isolates), comprised one cluster (SIT34/MIT311) with three isolates and two clusters (SIT71 and an orphan) with two isolates each, while Family 33 comprised three isolates and Family 36 one isolate. One EAI1 isolate and another Haarlem isolate were identified (Table 1).

## 4. Discussion

In a country like South Africa with high burden of TB, studies determining the population structure of TB strains in different geographical areas are important to monitor transmission. Information regarding MTB strains circulating in the Free State, situated in the centre of SA with little influx of people, is lacking. The only data available is from only one previous study including few isolates thus not representative of MTB strains in Free State [5]. The present study revealed high diversity of strains with three predominant lineages, namely, the LAM, T, and X families. Similarly, Stavrum et al., who were the first to report typing data from Free State, found diverse population of strains and the T lineage was the most prominent among 25 isolates from the province [5].

The diversity of MTB strains in the Western Cape [9, 10], KwaZulu-Natal [13, 14], Gauteng [11, 12], Mpumalanga, North-West, and Limpopo [24] has been described previously. In all these provinces the Beijing lineage was described as one of the predominant strains. In our study the LAM (33%) and T (31%) strain families were the predominant. These two families were also the most prevalent genotypes in Eastern Cape and KwaZulu-Natal [5]. The LAM and X lineages occurred in all the provinces, but the highest frequencies of the X lineage were in the Western Cape and Northern Cape [5]. In North-West and Limpopo the EAI1_SOM strains, which originated in Somalia [17] and are present in Europe, Asia, and the Middle East, predominated. It is further present in high numbers in Gauteng and Mpumalanga, while our study cohort contained one of these strains [24].

In this study, the diversity within the LAM family was lower (56.0%) as compared to the T and X families. The largest clone in our study, SIT33/LAM3/MIT213 as demonstrated by both spoligotyping and MIRU-VNTR typing, missed spacers 9–11 (Figure 1). It seems that this strain is well adapted to the Free State. A similar subfamily was reported as the F11/SIT33 strain in the Western Cape where it is highly successful [25].

The deadly KZN/F15/LAM4 strain from KwaZulu-Natal differs by only one spacer from our isolates GF27 and ZT48, which miss spacers 39 and 41, respectively, instead of spacer 40 as the KwaZulu-Natal XDR-TB strain [14, 26]. In Zimbabwe, 32% of MTB isolates are reported as LAM-ZWE variants with the LAM-11-ZWE variant (SIT1468) present in the largest cluster among MDR-TB strains [27, 28]. Two of the isolates in our study, Q20 and ZT08, were identified as SIT813/LAM11_ZWE and SIT2196/LAM11-ZWE, respectively (Table 1). Both these variants were found in low numbers among MDR-TB isolates in Zimbabwe [28]. Comparing our results to what has been reported for other African countries shows that this family is circulating throughout Africa [22].

Comparison of our isolates to international strains on the MIRU-VNTRplus database using the neighbour-joining algorithm to obtain a phylogenetic comparison showed that seven of our SIT53/T1 isolates grouped together in a cluster of 10 isolates from Ghana. These isolates from Ghana had identical spoligotypes to our strains [22]. Nine SIT53/T1 isolates (8.57%) and three SIT119/X1 isolates were reported in a study of 105 isolates from Ethiopia. Although these were the only isolates in our study that correlated with the Ethiopian isolates, they are also the most prevalent globally [29].

Strains from the X family are characterized by absence of spacers 5–12, especially in the X3 subfamily, as shown in Figure 1. ST119/X1 and SIT92/X3 strains found in this study have been reported in Guadeloupe [30], the Anglo-Saxon countries [31], and the Western Cape [32], and they are highly prevalent in KwaZulu-Natal [14], given the fact that both the X3 and Beijing strains are less prevalent in the Free State compared to the Western Cape. The Beijing types have been characterized extensively due to reported association with drug resistance and global dissemination [33–36]. Our study included five Beijing strains, three from the Thabo Mofutsanyane district, two from Mangaung, and none from Lejweleputswa. Three of the Beijing strains in our study came from the same clinic indicating possible transmission. All three belonged to the W type, which caused a notorious outbreak in New York at the beginning of the 1990s [37]. The same strain is also present in great numbers in the Western Cape [38], Gauteng [11], and was reported by Stavrum et al. as the second most prevalent lineage in SA [5]. Even though our isolates were susceptible strains with few monoresistant, they reflect a low multidrug-resistant TB (MDR-TB) burden in our province. Other studies from South Africa that included susceptible strains and monoresistant strains like our study did not find any difference in genotypes between MDR-TB and susceptible strains [39, 40].

Although 10 strains with isoniazid resistant phenotypes were included in this study, these were widely dispersed among the genotypes with no association between them.

This study was limited by small sample size and noninclusion of MDR-TB and XDR-TB isolates.

## 5. Conclusions

There is extensive strain diversity of MTB strains in the Free State province. This may indicate nonclonal transmission with diverse strains contributing to TB dynamics.

There remains a need to type current isolates to get a clear understanding on the genotypic population structure of MTB strains and the transmission dynamics of drug- resistant strains.

## Figures and Tables

**Table 1:** Distribution of the various *M. tuberculosis* SITVIT and presumptive SPOTCLUST combined with the randomised initialising model of the TB-lineage/TB-lineage database families present in the three areas of the Free State province of South Africa.

FS district	Number of isolates	Spoligotype family with number of isolates and the number of isolates with a SIT number, in brackets																												
		SPOTCLUST/RIM	LAM1	LAM3		LAM8	LAM9			T1					T2		T4	T5-RUS	S		X1	X3		Beijing	EAI1	Family 33			Family 36	H1
		TB-lineage	LAM1	LAM3	AFRI	LAM11-ZWE	LAM	LAM4	T5-RUS1	T	T2	T3	T5	T2-Uganda	X2	S	T5-RUS1	T5-RUS	S	H3-Ural-1	X1	X3	BOV_3	Beijing	CAS	EAI1_SOM	T3-OSA	Many1	T1-RUS2	H1
Lejweleputswa	22			6 (4)		1 (1)		1		4 (3)	1								1			3 (2)		3 (3)		1	1			
Thabo Mofutsanyane	23		1	6 (4)			2 (2)			3 (2)		1	2 (1)	1		1		1 (1)	1			2 (2)	1		1					
Mangaung	41			7 (5)	1	1 (1)	1 (1)		1	11 (6)					1		1 (1)		8 (6)	1	2 (1)			2 (2)				1	1	2 (1)

	86		1	19 (13)	1	2 (2)	3 (3)	1	1	18 (11)	1	1	2 (1)	1	1	1	1 (1)	1 (1)	10 (6)	1	2 (1)	5 (4)	1	5 (5)	1	1	1	1	1	2 (1)
